# Assessment and validation of three spot urine assay methods for the estimation of 24‐hour urinary sodium excretion in Chinese Tibetan adults living in the mountains

**DOI:** 10.1111/jch.14312

**Published:** 2021-07-01

**Authors:** Xin Zhang, Hang Liao, Runyu Ye, Xinran Li, Qiling Gou, Zhipeng Zhang, Rufeng Shi, Qingtao Meng, Zewong Zhuoma, Hengyu Zhang, Xiaoping Chen

**Affiliations:** ^1^ Department of Cardiology West China Hospital Sichuan University Chengdu Sichuan Province People's Republic of China; ^2^ Luohuo County Health Bureau Ganzi Tibetan Autonomous Prefecture Luhuo Sichuan Province People's Republic of China

**Keywords:** INTERSALT, Kawasaki, salt, Tanaka, Tibetan, urinary sodium excretion

## Abstract

Twenty‐four‐hour urine collection is the gold standard method for the evaluation of salt intake, but it is often impractical in large‐scale investigations, especially in resource‐poor areas. Methods for the estimation of 24‐hour urinary sodium excretion (USE) using a spot urine sample have been established, but have not been validated in Chinese Tibetans. Therefore, the authors aimed to evaluate the Kawasaki, Tanaka, and the International Cooperative Study on Salt, Other Factors, and Blood Pressure (INTERSALT) formulas for the prediction of 24‐hour USE in Chinese Tibetan adults. The authors analyzed the bias, correlation, agreements between estimated values and measured values, and the relative and absolute differences and misclassification at the individual level for the three methods in 323 Tibetan participants from the Ganzi Tibetan Autonomous Prefecture of Sichuan Province, China. The mean biases between the measured values and the estimated 24‐hour USE using the Kawasaki, Tanaka, and INTERSALT methods were 5.4 mmol/day (95% confidence interval [CI]: 0.8–10.1 mmol/day), −40.8 mmol/day (95% CI: −44.6 to −36.9 mmol/day), and −57.1 mmol/day (95% CI: −61.9 to −52.4 mmol/day), respectively. The Pearson correlation coefficients for the relationships between the measured values and the estimated 24‐hour USE were 0.43 (Kawasaki), 0.38 (Tanaka), and 0.27 (INTERSALT), respectively (all *p *< .01). The intraclass correlation coefficients showed similar patterns to the correlation data: 0.47 for Kawasaki, 0.40 for Tanaka, and 0.27 for INTERSALT (all *p *< .01). The upper and lower limits of agreement between the measured values and the estimated 24‐hour USE were −92.6 and 81.8 mmol/day for the Kawasaki method, −28.5 and 110.0 mmol/day for the Tanaka method, and −28.4 and 142.7 mmol/day for the INTERSALT method. Compared with the other two methods, the percentage of individuals that were misclassified by using the Kawasaki method was 48.2%, while those for the Tanaka and INTERSAL methods was 72.1% and 75.5%, respectively. However, when an individual's salt intake was higher than 12.8 g/day, the misclassification rates of the Kawasaki, Tanaka, and INTERSALT methods were 20%, 90%, and 97.5%, respectively. Thus, the authors found that the Kawasaki equation may have performed better than the other equations at Chinese Tibetan population level assessment, but none of these equations are suitable for use or perform well at the individual level. A more accurate method of using a spot urine sample to evaluate individual 24‐hour USE for Tibetans is needed.

## 1 INTRODUCTION

High salt intake is an important and preventable risk factor for hypertension. The International Cooperative Study on Salt, Other Factors, and Blood Pressure (INTERSALT) confirmed that individual salt intake was closely related to blood pressure. Specifically, for every 100 mmol increase in 24‐hour urinary sodium excretion (USE), systolic and diastolic blood pressure increased by 6.0 and 2.5 mmHg, respectively.^1^ In addition, high salt intake can cause target organ damage,^2^, ^3^ including left ventricular hypertrophy, renal functional impairment, arterial stiffness, and high urinary albumin/creatinine ratio, which significantly increase the risk of cardiovascular events.^4^ Of the global number of deaths owing to cardiovascular diseases, approximately 1.65 million per year have been attributed to excessive salt intake.^5^

Tibetans are the eighth largest ethnic minority in China, with a population of more than 6 million. A previous epidemiologic survey demonstrated that the prevalence of hypertension in Tibetans ranked front among the 56 ethnic groups in China.^6^ This heavy burden of hypertension has been related to the high altitude at which they live, with low atmospheric pressure predisposing toward hypoxia^7^; genetic factors^8^; and the special lifestyle of Tibetan residents, such as the consumption of salty beverages and cured yak meat. Therefore, a survey of salt intake in Tibetans may be particularly important for the prevention and treatment of hypertension in this area.

The accurate assessment of salt intake is an essential prerequisite for the design of strategies aimed at reducing salt consumption. The existing methods of measuring salt intake include dietary recall, weighted diet records, food frequency questionnaires, 24‐hour urine collection, and spot urine sample collection.^9^ Of these, 24‐hour urine collection is recognized as the “gold standard” method for the evaluation of salt intake,^9^ but the collection process is time‐consuming, expensive, troublesome, and complex for participants.^10^ In addition, incomplete emptying of the bladder, poor storage of the samples, and incorrect or incomplete collection might generate errors that significantly affect its accuracy.^10^ Therefore, 24‐hour urine collection is challenging in large‐scale epidemiological investigations, and especially in those conducted in remote and/or resource‐poor areas. In comparison, spot urine sample‐based methods are relatively simple and inexpensive, with the collection and storage of samples being easier. Therefore, they may be practical and affordable alternatives to 24‐hour USE for use in population surveys.^11^ The spot urine methods for estimation of USE that commonly used are the Kawasaki formula,^12^ Tanaka formula,^13^ and INTERSALT formula.^14^ However, the results of recent studies on the validity and reliability of spot urine sample‐based methods for the estimation of 24‐hour USE have yielded contradictory findings.^15^, ^16^ Moreover, the use of these formulas has not been validated in Chinese Tibetans.

Therefore, the aim of the present study was to compare the use of the Kawasaki, Tanaka, and INTERSALT methods for the estimation of 24‐hour USE in a general population of Chinese Tibetan adults living in the mountains. We anticipate that this study would lay a foundation for estimating and monitoring salt intake in Chinese Tibetans.

## 2 METHOD

### 2.1 Study participants

We performed a cross‐sectional study of adults over 18 years old who were recruited in Jiulong and Luhuo Counties, Ganzi Tibetan Autonomous Prefecture, Sichuan Province. The inclusion criteria were as follows: (1) Tibetans ethnicity; (2) >18 years old; and (3) voluntary participation, with the provision of written informed consent. The exclusion criteria were as follows: (1) vision or hearing impairment, or physical disability; (2) unwillingness to cooperate or provide written informed consent; (3) severe mental illness; (4) pregnancy, planned pregnancy, or lactation during the study period; (5) menstruation during the study period; (6) dietary restrictions or disorders caused by a chronic disease, such as end‐stage kidney disease, congestive heart failure, malignant tumors, cachexia, or acquired immunodeficiency syndrome; (7) long‐term oral administration of drugs that might cause metabolic disorders of sodium or potassium, including furosemide, spironolactone, hydrochlorothiazide, angiotensin‐converting enzyme inhibitors, angiotensin II receptor antagonists, methyldopa, and oral contraceptive; (8) diseases that cause abnormal renal sodium excretion or sodium retention, including cerebral salt wasting syndrome, abnormal antidiuretic hormone secretion syndrome, 11β or 17 α‐hydroxylase deficiency, Bart syndrome, and aldosteronism; (9) acute or chronic urinary tract infection; (10) chronic vomiting, diarrhea, or cirrhotic ascites; and (11) inability to complete 24‐hour urine and morning urine collection within the specified time period. The study was conducted according with the principles of the Declaration of Helsinki guidelines,^17^ and all the procedures involving human were approved by the ethics committee of West China Hospital, Sichuan University.

### 2.2 Questionnaire investigation and physical examination

Version 1.0 of the hypertension research questionnaire for residents in Ganzi Prefecture, identified by the experts of the project team, was used in this survey. Doctors and nurses who accepted the uniformly training were arranged to participate in field investigation, equipped with a Tibetan interpreter to ensure smooth communication and the quality of the original data. The contents of the questionnaire covered general information, past disease status, present illness, medication use, family history, personal lifestyles, etc. Height, weight, waistline, hip circumference, and blood pressure were measured in a room with a temperature of about 25°C. In this study, we utilized the verified electronic sphygmomanometer (OMRON HBP‐1100, OMRON Healthcare, Kyoto, Japan) to obtain the participants’ blood pressure value. Sitting position blood pressure on the right arm was consecutively measured three times with 1‐minute interval for each participant. The average value of the three readings was used for final analyses.

### 2.3 Collection and measurement of urine samples

#### 2.3.1 Methods of collection

All the participants were asked to maintain their normal diet and liquid intake, and to avoid a high‐protein diet and strenuous physical activity. Urine collection was performed on 2 consecutive days (days 1–2): 24‐hour urine collection was performed on day 1 and a second morning urine (SMU) sample was collected on day 2. To ensure a complete urine collection, written and oral instructions were given to the participants. A 5‐L carrier with a lid and test tubes were given to each participant for 24‐h urine collection and the SMU sample, respectively.

For 24‐h urine collection, the participants were asked to discard their first voided urine after getting up, at approximately 07:00 a.m. (taking 7 o'clock am as an example) on day 1, and to collect all the urine they voided during the subsequent 24 hours, until 07:00 a.m. the following day, including urine initially voided on day 2. The volume of the 24‐hour urine was measured by the participant or a family member at home. A 5‐ml sample from the 5‐L container was decanted into a test tube for analysis after stirring. The SMU sample was collected before breakfast after the first voiding of urine on the morning of day 2. The participants were asked to transfer a portion of their SMU into the test tube for further testing. The participants were asked to record the start and finish times of the collections, the 24‐hour total urine volume, the times of urine leakage, their physical activities, and any medication used on a record sheet.

All the samples were stored at −80°C and transported to the Department of Experimental Medicine, West China Hospital of Sichuan University, under the protection of liquid nitrogen or dry ice. The urine sodium and potassium concentrations were determined using the electrode method, and the urine creatinine concentration was determined using the enzymatic method. A complete 24‐hour urine collection was defined as urine volume ≥ 500 ml, a recorded collection time ≥ 20 hours, and no report of spillage or omission of the collection of urine more than once during the 24‐hour period.^18^, ^19^ Participant data were excluded if the standard protocol for the collection of urine samples was not followed. Contamination of samples, resulting for example from poor storage and the growth of urine alkalizing bacteria,^10^ or inclusion of fecal matter, also resulted in the exclusion of sample data from the analyses. In addition, overcollection (urinary creatinine or volume > 3 times SD of the population mean) were also excluded for the final analysis.^20^

#### 2.3.2 Methods of evaluating urine sodium excretion

The methods for the estimation of 24‐hour USE by using spot urine samples were Kawasaki method,^12^ Tanaka method,^13^ and INTERSALT method.^14^ The estimation formulas used are listed in Table 1. The measured 24‐hour USE (mmol/day) =
24‐hour urinary sodium concentration (mmol/L)×the volume of the 24‐hour total urine sample (L/day). Salt intake (mg/day) = 2.54 × 23 ×24‐hour USE (mmol/day).

**TABLE 1:** Equations to estimate 24‐hour excretion rate by spot urine sodium method included in this study

Method	Equations to estimate 24‐hour urinary sodium excretion rate (mmol/day)
Kawasaki^11^	PrUCr_24h_ (female) = −4.72*age +8.58*weight (Kg)+5.09*height (cm) − 74.5
	PrUCr_24h_ (male) = −12.63*age +15.12*weight (Kg)+7.39*height (cm) − 79.9
	Estimation of 24‐hour USE = 16.3* (Na_spot_ (mmol/L)/Cr_spot_ (mg/dl)) *0.1*PrUCr_24h)_^0.5^
Tanaka^12^	PrUCr_24h_ = [−2.04* age +14.89* weight (Kg)+16.14* height (cm) − 2244.45]
	Estimation of 24‐hour USE = 21.98[Na_spot_ (mmol/L)/Cr_spot_ (mg/dl)* PrUCr_24h_]^0.392^
INTERSALT^13^	Estimation of 24‐hour USE (female) = {5.07+[0.34* Na_spot_ (mmol/L)]‐[2.16* Cr_spot_ (mmol/L)]‐[0.09* K_spot_ (mmol/L)]+[2.39*BMI (Kg/m^2^)]+[2.35* age]‐[0.03* age^2^]}
	Estimation of 24‐hour USE (male) = {25.46+[0.46* Na_spot_ (mmol/L)]‐[2.75* Cr_spot_ (mmol/L)]‐[0.13* K_spot_ (mmol/L)]+[4.10*BMI (Kg/m^2^)]+[0.26* age]}

Abbreviations: BMI, body mass index; Cr_spot_, spot urinary creatinine; INTERSALT, International Cooperative Study on Salt, Other Factors, and Blood Pressure; K_spot_, spot urinary potassium; Na_spot_, spot urinary sodium; PrUCr_24h_, predicted 24‐h urinary creatinine; USE, urinary sodium excretion.

### 2.4 Related definition

Hypertension was defined by the average systolic blood pressure ≥ 140 mmHg and/or diastolic blood pressure ≥ 90 mmHg at the physical examination or who were receiving the antihypertensive therapy or who had a previous diagnosis of systemic hypertension before this investigation.^21^ Diabetes mellitus (DM) was defined by a previous diagnosis of type 1 and 2 DM with or without hypoglycemic therapy or fasting venous blood glucose ≥ 7.0 mmol/L this time.^22^ The formula of body mass index was as follow: weight (kg)/height (m^2^).

### 2.5 Statistic methods

SPSS 23.0 statistical software (IBM, Inc., Armonk, NY, USA) was used for the analyses. Continuous data are expressed as means ± SDs and categorical variables are expressed as frequency (%). Paired t‐test was utilized to evaluate the differences between the estimated values and measured values. Bivariate correlation analysis and intraclass correlation coefficients (ICCs) were used to evaluate the relationships between the estimated values and the measured values of 24‐hour USE. Bland–Altman plots were also used to evaluate the agreement between the estimated and measured values of 24‐hour USE. A regression line fitted to the mean 24‐hour USE and the difference in 24‐hour USE by two methods to examine whether the bias between the estimated and the measured value was proportional to the level of sodium excretion. In addition, we analyzed the relative and absolute differences between the estimated and measured values, where the relative difference was calculated as [(estimated value − measured value)/measured value × 100%] and the absolute difference was calculated as (estimated value − measured value). The proportional distribution of the relative and absolute differences provided a visualized assessment of the accuracy at the individual level. The relative difference in this study was defined in five groups: within ±10%, ±10% to 19%, ±20% to 29%, ±30% to 39%, and over ±40%. The absolute difference was also divided into five groups that were within ±17.1, ±17.1 to 34.2, ±34.2 to 51.3, ±51.3 to 85.5, and over ±85.5 mmol/day in sodium amount (equivalent to ±1, ±1 to 2, ±2 to 3, ±3 to 5, and over ±5 g/day in salt amount). Using the approach of Zhou and colleagues,^23^ we converted the estimated and measured 24‐hour USE values into salt intakes, divided these salt intakes into quartiles according to the measured 24‐hour USE (<10, 10–11.4, 11.5–12.7, and ≥12.8 g/day), and compared the proportions of the participants who were placed into each same group using each method. *p *< .05 was considered to represent statistical significance.

## 3 RESULTS

### 3.1 Characteristics of the participants

**FIGURE 1:**
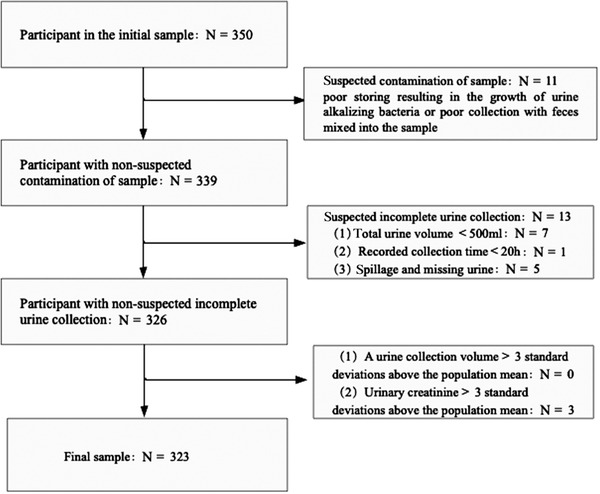
Flow diagram of the participants included in the final sample for statistical analysis

Three hundred fifty participants were initially recruited, of whom 13 with incomplete 24‐hour urine collection, 11 with suspected urine sample contamination, and 3 whose 24‐hour urinary creatinine > 3 times SD of the population mean were excluded (Figure 1). Therefore, data for 323 participants were included in the analyses. The baseline characteristics of the participants are presented in Table 2. Of the participants, 61% were females, and 54.5% and 5% had been diagnosed with hypertension and DM, respectively. They were 51.2 ± 15.1 years old. The mean ± SD sodium concentration of their 24‐hour urine samples was 125.8 ± 21.5 mmol/L and their mean ± SD 24‐hour urine volume was 1539.7 ± 195.6 ml. The mean concentrations of sodium, potassium, and creatinine in their spot urine samples were 151.5, 55.6, and 11.2 mmol/L, respectively.

**TABLE 2:** Baseline clinical data of participants in this study

Variables (N = 323)	Value
Age (years old)	51.2±15.1
Female (%)	197 (61%)
Height (cm)	160.2±8.7
Weight (kg)	64.4±11.5
BMI (kg/m^2^)	25.0±3.8
WC (cm)	85.9±12.0
HC (cm)	93.2±7.8
SBP (mmHg)	136.1±22.5
DBP (mmHg)	84.1±13.7
HR (rate/min)	79.6±13.6
Hypertension (%)	176 (54.5%)
DM (%)	16 (5%)
Second morning urinary sample	
Sodium concentration (mmol/L)	151.5±39.0
Potassium concentration (mmol/L)	55.6±22.8
Creatinine concentration (mmol/L)	11.2±4.4
24‐Hour urinary sample	
Sodium concentration (mmol/L)	125.8±21.5
24‐Hour urine volume (ml)	1539.7±195.6

Abbreviations: BMI, body mass index; DBP, diastolic blood pressure; DM, diabetes mellitus; HC, hip circumference; HR, heart rate; SBP, systolic blood pressure; WC, waist circumference.

### 3.2 Measured versus estimated 24‐hour USEs

The mean ± SD measured 24‐hour USE of the participants was 198.8 ± 38.7 mmol/day, and it was higher in men than in women (201.6 ± 48.4 mmol/day vs 197.0 ± 31.5 mmol/day, respectively; *p <* .01). The differences between the measured and estimated 24‐hour USE values are shown in Table 3. Of the three methods, the Kawasaki method overestimated the measured values by 24‐hour urine sample and the mean bias was 5.4 mmol/day (95% confidence interval [CI]: 0.8–10.1 mmol/day). In contrast, the values estimated using the Tanaka, and INTERSALT methods were all lower than the measured values by 24‐hour urine sample. The mean differences between the values estimated using the Tanaka and INTERSALT methods and the measured value by 24‐hour urine sample were −40.8 mmol/day (95% CI: −44.6 to −36.9 mmol/day) and −57.1 mmol/day (95% CI: −61.9 to −52.4 mmol/day), respectively.

**TABLE 3:** Comparison between the estimated 24‐hour urinary sodium excretion rate by the four spot sodium methods and the measured 24‐hour urinary sodium excretion (mmol/day)

Categories	Measured 24‐hour USE	Estimated 24‐hour USE by Kawasaki	Estimated 24‐hour USE by Tanaka	Estimated 24‐hour USE by INTERSALT
All participants	198.8±38.7	204.2±47.5^#^	158.0±29.6^*^	141.7±33.3^*^
Male (N = 126)	201.6±48.4	217.9±49.4^*^	158.2±29.9^*^	170.0±26.3^*^
Female (N = 197)	197.0±31.5	195.0±44.2	157.9±29.4^*^	123.8±23.4^*^
Mean difference	Ref.	5.4	−40.8	−57.1
95% CI mean difference	Ref.	(0.8, 10.1)	(−44.6, −36.9)	(−61.9, −52.4)

Estimated 24‐hour urinary sodium excretion rate by Kawasaki, Tanaka, INTERSALT, SH2 method were compared with measured 24‐hour urinary sodium excretion.

Abbreviations: INTERSALT, International Cooperative Study on Salt, Other Factors, and Blood Pressure; USE, urinary sodium excretion.

The estimated values compared with measured values, *P* < .05 was statistically significant; ^*^ *P* < .001;^#^ *P* < .05.

### 3.3 Relationships between the measured and estimated 24‐hour USE values

The Pearson correlation coefficients for the relationships between the measured values and the estimated 24‐hour USE values calculated using the various methods (the Kawasaki, Tanaka, and INTERSALT methods) were 0.43 (*P <* .01), 0.38 (*P <* .01), and 0.27 (*P <* .01), respectively (Table 4). The ICCs showed a similar pattern to the results of the correlation analysis, 0.47 (95% CI: 0.38–0.55) for the Kawasaki method, 0.40 (95% CI: 0.25–0.47) for the Tanaka method, and 0.27 (95% CI: 0.17–0.37) for the INTERSALT method (all *p <* .01). (Table 4).

**TABLE 4:** Comparison the correlation between the estimated 24‐hour urinary sodium excretion rate by the four spot sodium methods and the measured 24‐hour urinary sodium excretion

Categories	Measured 24‐hour USE	Estimated value by Kawasaki	Estimated value by Tanaka	Estimated value by INTERSALT
Pearson coefficient	Ref.	0.43*	0.38*	0.27*
ICC (95% CI)	Ref.	0.47	0.40	0.27
		(0.38–0.55)	(0.25–0.47)	(0.17–0.37)

Correlation and reliability comparison between the estimated values by Kawasaki method, Tanaka method, INTERSALT method, and SH2 method and the measured values by 24‐hour urinary sodium method.

Abbreviations: ICC, intraclass correlation coefficient; INTERSALT, International Cooperative Study on Salt, Other Factors, and Blood Pressure; USE, urinary sodium excretion.

* *P* < .01.

### 3.4 Agreements between the measured and estimated 24‐hour USE values

**FIGURE 2:**
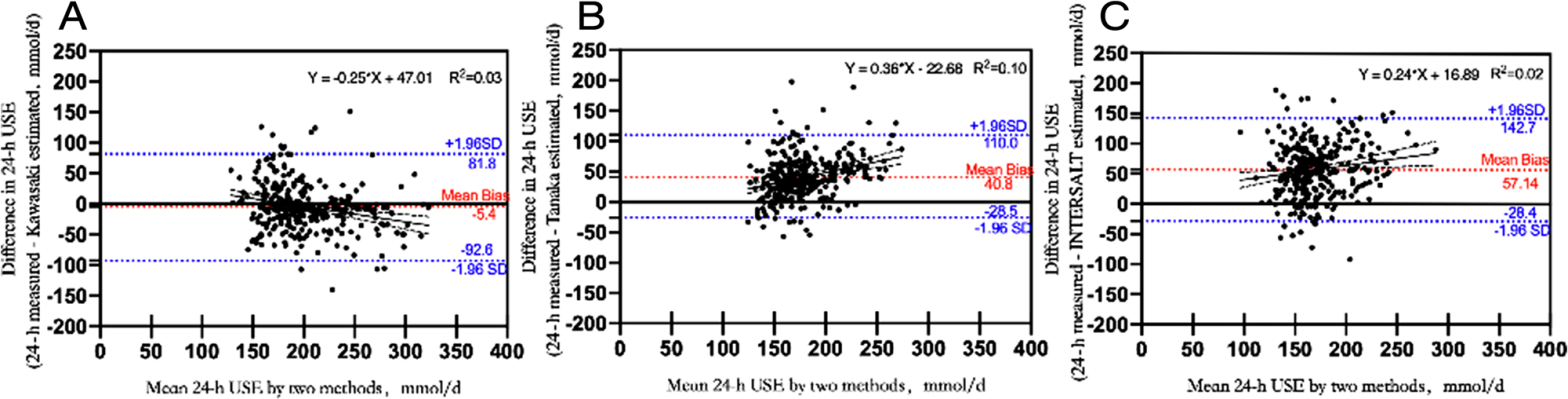
Bland–Altman plots of the measured versus estimated 24‐hour USE. (A) Kawasaki method, (B) Tanaka method, and (C) INTERSALT method. The differences were calculated as the measured values minus the estimated values; the upper and lower limits of agreement (blue dashed lines) are equal to the mean difference ± 1.96 × SD of the difference; the red‐dashed line represents the mean difference/bias between the measured and estimated values; regression line is represented by the black solid line; the black dashed lines are the 95% confidence interval for the slope of the fitted curve. USE, urinary sodium excretion

Bland–Altman plots were used to evaluate the agreement between the values estimated using the three methods and the measured 24‐hour USE values, and the results are shown in Figure 2. The Kawasaki method overestimated the measured value, whereas the Tanaka, and INTERSALT methods underestimated the measured value to varying extents. Of the three methods, the Kawasaki method provided the least biased population estimation of 24‐hour USE. The upper and lower limits of agreement between the estimated values and measured 24‐hour USE values were −92.6 and 81.8 mmol/day for the Kawasaki method (Figure 2A), −28.5 and 110.0 mmol/day for the Tanaka method (Figure 2B), and −28.4 and 142.7 mmol/day for the INTERSALT method (Figure 2C). In addition, we used a regression line fitted to the mean and the difference in 24‐h USE by two methods to examine whether the bias between the estimated and the measured value was proportional to the level of sodium excretion. It showed that the fitted curve equation was Y = −0.25*mean 24‐hour USE + 47.01 (R^2 ^= 0.03) in Figure 2A (Kawasaki method vs 24‐hour urine collection method), Y = 0.36*mean 24‐hour USE − 22.68 (R^2 ^= 0.10) in Figure 2B (Tanaka method vs 24‐hour urine collection method), and Y = 0.24*mean 24‐hour USE + 16.89 (R^2 ^= 0.02) in Figure 2C (INTERSALT method vs 24‐hour urine collection method), respectively. 95% CI for the slope of the fitted curve was −0.37 to −0.14 (*P* < .01) in Figure 2A, 0.23–0.48 (*P* < .01) in Figure 2B, and 0.07–0.40 (*P* < .01) in Figure 2C, respectively.

### 3.5 Relative and absolute differences between the measured and estimated 24‐hour USE values

**FIGURE 3:**
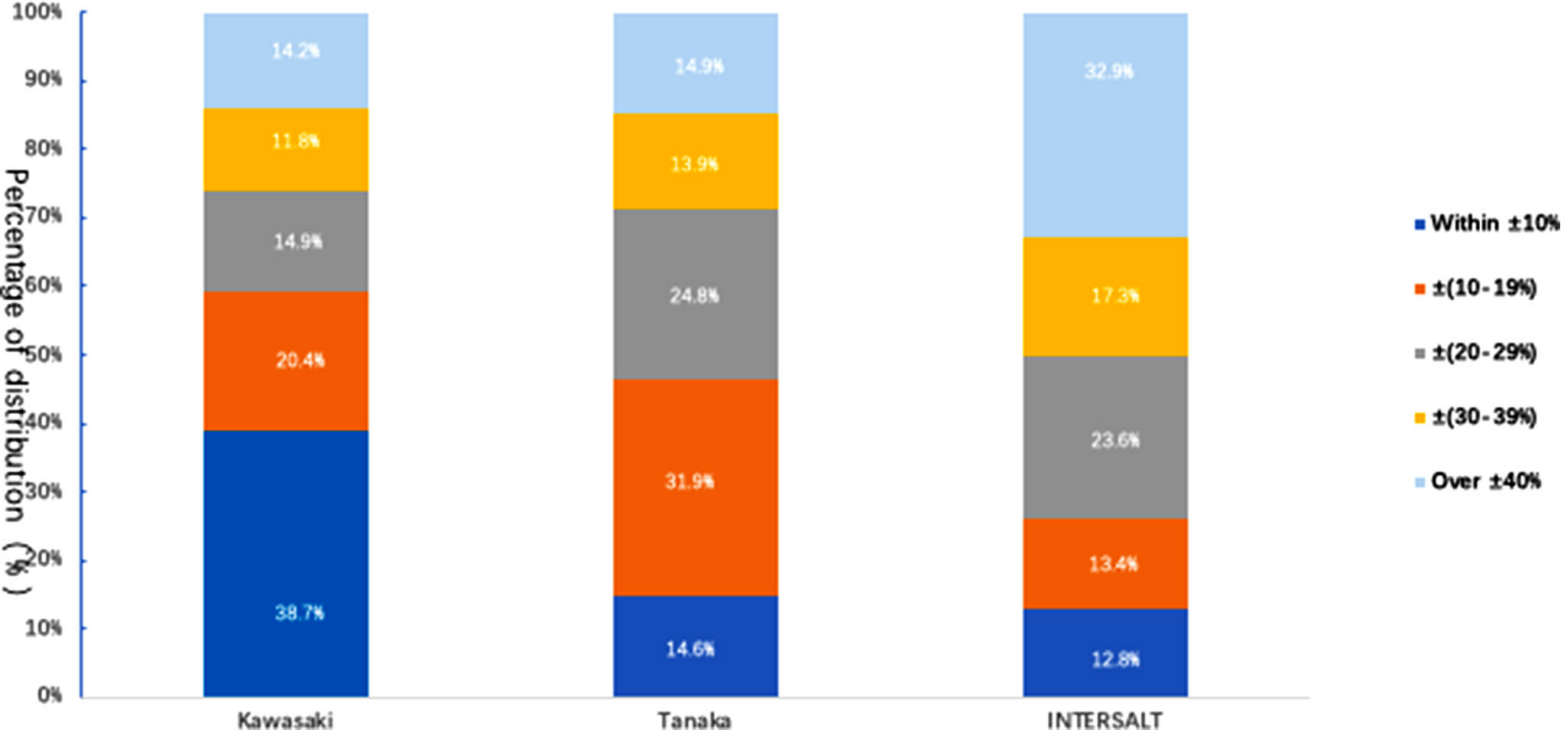
Relative difference distributions for the Kawasaki, Tanaka, and INTERSALT methods for the estimation of sodium intake. Relative difference = [(estimated value − measured value)/measured value × 100%]

**FIGURE 4:**
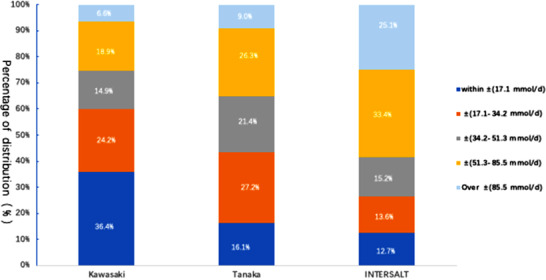
Absolute difference distributions for the Kawasaki, Tanaka, and INTERSALT methods for the estimation of sodium intake. Absolute difference = (estimated value‐measured value)

When using the measured 24‐hour USE as the reference, the proportions of the samples for which the relative differences were within ±10% for the Kawasaki, Tanaka, and INTERSALT methods were 38.7%, 14.6%, and 12.8%, respectively. The proportions of samples for which the relative differences were identified that were greater than ±40% was 14.2%, 14.9%, and 32.9%, respectively. The proportions of the samples for which the individual absolute differences were within ± 17.1 mmol/day (approximately salt intake = 1 g/day) for the Kawasaki, Tanaka, and INTERSALT methods were 36.4%, 16.1%, and 12.7%, respectively. Finally, the proportions of the individual absolute differences that were over ±85.5 mmol/day (approximately salt intake = 5 g/day) were 6.6%, 9.0%, and 25.1%, respectively. Figures 3 and 4 summarize these findings.

### 3.6 The percentage of individuals that were misclassified using the three methods for the assessment of salt intake

We also compared the individual salt intake classification group according to their measured salt intake compared with estimated value by using three formulas (Table 5). According to the quartile of measured 24‐hour USE (<172.50, 172.51–194.50, 194.51–218.94, and ≥218.95 mmol/days), the participants were allocated to four sub‐groups, corresponding to salt intakes < 10, 10–11.4, 11.5 and 12.7, and ≥12.8 g/day, respectively. The results showed that the percentage of individuals that were misclassified when using the Kawasaki method was 48.2%, and those for the Tanaka and INTERSALT methods were 72.1% and 75.5%, respectively. Furthermore, with the increases in individual salt intake, the percentage of individuals that were misclassified when using the Kawasaki method gradually decreased. When an individual's salt intake was higher than 12.8 g/day, the percentage of individuals that were misclassified by using the Kawasaki, Tanaka, and INTERSALT methods were 20%, 90%, and 97.5%, respectively.

**TABLE 5:** Misclassification of the three estimation methods for individual salt intake

	Salt intake was converted according to the measured 24‐hour urinary sodium excretion rate				
Method/categories	<10 g (N = 81)	10–11.4 g (N = 80)	11.5–12.7 g (N = 82)	≥12.8 g (N = 80)	Total (N = 323)
Kawasaki method					
<10 g	29 (35.8%)	19 (23.8%)	9 (11.0%)	11 (13.75%)	
10–11.4 g	30 (37.0%) )	32 (40%)	6 (7.3%)	3 (3.75%)	
11.5–12.7 g	21 (25.9%)	29 (36.2%)	42 (51.2%)	2 (2.5%)	
≥12.8 g	1 (1.3%)	0 (0%)	25 (30.5%)	64 (80%)	
Misclassification	52 (64.2%)	48 (60%)	40 (48.8%)	16 (20%)	156 (48.2%)
Tanaka method					
<10 g	74 (91.4%)	74 (92.5%)	59 (72%)	22 (27.5%)	
10–11.4 g	5 (6.2%)	4 (5.0%)	16 (19.5%)	31 (38.8%)	
11.5–12.7 g	2 (2.4%)	2 (2.5%)	4 (4.9%)	19 (23.7%)	
≥12.8 g	0 (0%)	0 (0%)	3 (3.6%)	8 (10%)	
Misclassification	7 (8.6%)	76 (95%)	78 (95.1%)	72 (90%)	233 (72.1%)
INTERSALT method					
<10 g	70 (86.4%)	74 (92.5%)	76 (92.7%)	47 (58.8%)	
10–11.4 g	8 (9.9%)	5 (6.3%)	4 (4.9%)	15 (18.7%)	
11.5–12.7 g	3 (3.7%)	1 (1.2%)	2 (2.4%)	16 (20%)	
≥12.8 g	0 (0%)	0 (0%)	0 (0%)	2 (2.5%)	
Misclassification	11 (13.6%)	75 (93.7%)	80 (97.6%)	78 (97.5%)	244 (75.5%)

Abbreviation: INTERSALT, International Cooperative Study on Salt, Other Factors, and Blood Pressure.

## 4 DISCUSSION

To the best of our knowledge, the present study is the first to compare the Kawasaki, Tanaka, and INTERSALT methods for the estimation of the 24‐hour USE in the Chinese Tibetan population living in the mountains. Firstly, the Kawasaki method overestimated the measured value, but showed the lowest mean bias and closer correlation compared with the Tanaka, and INTERSALT methods. Secondly, all the equations performed poorly at the individual level, with wide limits of agreement and relatively high percentage of individuals that were misclassified. We deem that all equations performed poorly in this cohort and are not suitable for use in this population.

In the present study, the Kawasaki method provided the least biased population estimation of 24‐hour USE. This finding is consistent with those of previous studies conducted in Chinese populations.^23^, ^24^, ^25^, ^26^ For example, Peng and colleagues studied rural and urban residents from Shanxi Province who were participating in the Prospective Urban Rural Epidemiology (PURE)‐China study, and found that the mean differences between the measured values and values estimated using the Kawasaki, Tanaka, and INTERSALT methods were −740.49, −2305.05, and −2797.39 mg/day.^24^ Other studies^23^, ^25^, ^26^ that were performed in the Chinese populations also demonstrated that the differences between the measured values and values estimated using the Kawasaki method were smaller than that those obtained when using the Tanaka or INTERSALT methods. In addition, a sub‐study of the PURE study, which was of 1083 participants aged 35–70 years who were recruited worldwide, obtained similar results.^15^ However, the mean differences between the estimated values and the measured values differed among studies and populations. For example, studies of Korean,^27^ Portuguese,^28^ and Iranian^29^ participants found that the differences between the measured and estimated values obtained using the Tanaka method were smaller than those obtained using the Kawasaki and INTERSALT methods. Furthermore, studies conducted in South African^30^ and the United States^31^, ^32^ found that the differences between the values estimated using the Kawasaki method and the measured values were the largest.

The inconsistency of these findings implies that ethnicity and dietary pattern might affect the choice of the optimal means of estimating 24‐hour USE. In addition, the Kawasaki and Tanaka methods were established during the studies of the Japanese population, which has a high salt intake,^12^, ^13^ and therefore these methods might potentially overestimate the salt intake of individuals consuming a western diet. Conversely, the INTERSALT method was developed using the relatively low salt intake of the European and American populations,^14^ and therefore might underestimate the 24‐hour USE of individuals consuming a relatively large amount of salt. In particular, salt intake is high in Asia, and especially in east Asia countries, such as China, Japan, and Korea,^33^ as well as in Tibet, China.^34^ Moreover, a study conducted in the general Brazilian adult population found that the use of the Kawasaki formula was appropriate only when the salt consumption was between 12 and 18 g/day.^35^ In the present study, the mean salt intake of the Tibetan participants was 12.6 g/day, which is significantly higher than the global mean, which might explain the relatively small differences between the measured values and estimated values using the Kawasaki method in this study.

The Pearson correlation coefficients for the relationships between the measured values and the estimated 24‐hour USE values were low‐to‐moderate, 0.43, 0.38, and 0.27, for the Kawasaki, Tanaka, and INTERSALT methods, respectively. In addition, ICC analysis yielded values of 0.47, 0.40, and 0.27, respectively. Taken together, these findings demonstrated that the correlation between the estimated value by Kawasaki method and the measured value were better than those of Tanaka and INTERSALT methods. In previous studies, the correlation coefficients for the relationships between the values estimated using the Kawasaki method and the measured values were 0.31–0.407 in Chinese studies,^23^, ^25^, ^26^, ^36^ and 0.21–0.54 in the European population^28^, ^37^; and the corresponding ICCs were 0.26–0.48 in China,^23^, ^25^, ^26^, ^36^ and 0.303–0.54 in the Europe.^28^, ^37^ The differences in the correlation coefficients obtained in the various surveys may be explained by variations in genetic background of the studied populations, differences in dietary salt intake, and the differences in the collection methods for urine spot samples. In the present study, SMU samples were collected before breakfast, after initial voiding, which is standard for the Kawasaki method, on the day on which the 24‐hour urine collection was completed. This might explain why the correlation coefficients and ICC values in the present study were relatively higher than those obtained in previous studies. Another study of 222 hypertensive patients in China yielded a correlation coefficient for the relationship between the values estimated using the Kawasaki method and the measured values of 0.6485 using SMU samples,^18^ which is similar to the coefficient obtained in our study.

Bland–Altman plots are a useful and widespread means of analyzing the agreement between two quantitative measurements by studying the mean difference and constructing limits of agreement,^38^, ^39^ which might help us to compare the alternative method against the gold‐standard method. In this study, we have shown the Kawasaki method provided the least biased population estimation of 24‐hour USE among the three formulas tested. This finding is similar to those of studies performed in Chinese Jiangxi population and in elderly people who were at high risk of stroke in Shaanxi Province.^23^, ^26^ Though, most of differences lay between the upper and lower limits of agreement, but the limits were widely spaced for the three methods, including the Kawasaki formula, which implies that there is a great deal of variation in differences between the estimated and measured 24‐hour USE values at the individual level. We further found that the Tanaka and INTERSALT method displayed a trend of proportional bias: the bias between the estimated and measured value increased as sodium excretion increased. While, the Kawasaki method displayed an opposite trend.

Furthermore, we compared the relative and absolute differences between the values estimated using the three methods and the measured 24‐hour USEs, and found that the proportions of participants for whom the relative differences were greater than ±40% between the estimated and measured values for the Kawasaki, Tanaka, and INTERSALT methods were 14.2%, 14.9%, and 32.9%, respectively. In addition, the proportions of participants for whom the absolute differences between the estimated and measured values were greater than 5 g/day were 6.6%, 9.0%, and 25.1%, respectively. This further illustrates there is the potential for large errors in the estimation of 24‐hour USE by using the INTERSALT method for Chinese Tibetan adults.

Previous studies have shown that the percentage of individuals that were misclassified on the basis of a spot urine sodium measurement were mostly between 50% and 72%.^23^, ^40^ In the present study, the percentage of individuals that were misclassified was >45%, but that associated with the Kawasaki method was lower than that associated with the other two methods, which was 48.2%. These findings from our study do not support the use of a spot urine measurement for the estimation of 24‐hour USE in individuals. However, we found that as the 24‐hour USE increased, the percentage of individuals that were misclassified by using the Kawasaki method decreased. When individual salt intake was >12.8 g/day, the percentage of individuals that were misclassified by using the Kawasaki method was 20%, which was significantly lower than that associated with Tanaka, and INTERSALT methods. This might imply that the Kawasaki method may be preferable for the assessment of 24‐hour USE in people with high salt intake.

The spot urine samples were collected on only one occasion, and therefore may be subject to random measurement biases related to timing of meals, exercise, the use of medication, and variations in urine sodium or osmolarity during a day or interday.^41^, ^42^ In addition, methods developed on the basis of spot urine sodium measurements in specific ethnic groups might be associated with systemic errors^.^ ^41^ Therefore, the use of spot urine samples to estimate 24‐hour USE is controversial. However, in comparison to the difficulty of collecting 24‐hour urine samples for use in large‐scale epidemic investigations, these spot urine methods are relatively simple, standardized, low‐cost methods, and they can be effective substitutes for the 24‐hour urine sodium method for the monitoring of salt intake. Of these methods, the Kawasaki has been suggested by various studies to provide a good approximation of 24‐hour USE on a population level, and can thus be used for monitoring changes in 24‐hour USE in salt reduction studies.^15^, ^43^

The present study had some limitations. First, the participants in this study were Sichuan Tibetans, and there are five Tibetan areas in China. Therefore, the applicability of the findings to the other areas remains to be determined. Second, urine collection was conducted by the participants rather than the trained investigators, which might affect the quality of this study in some degree. Third, only one spot urine and one 24‐hour urine sample were collected. To minimize the impact of variations in daily salt intake among individuals on the bias, relationship, and agreement between measured and estimated values, 24‐hour and SMU samples could have been collected on several consecutive days. In addition, the circadian variation in urinary sodium and creatinine excretion means that single spot urine sample collected in the morning does not precisely represent the sodium excretion over a whole day. Some researchers have advocated the use of afternoon or evening spot samples, which would better represent 24‐hour USE, to evaluate salt intake. We intend to assess the accuracy of the use multiple spot urine samples or their combination, including SMU, random urine, and afternoon or evening spot samples to estimate 24‐hour USE, in future studies. Fourth, we excluded the hypertensive patients who were taking drugs that may cause metabolic disorders of sodium and potassium, including furosemide, spironolactone, hydrochlorothiazide, angiotensin‐converting enzyme inhibitors, angiotensin II receptor antagonists, and methyldopa, as well as oral contraceptive. Further larger‐scale studies should be conducted in Chinese Tibetan patients who are taking antihypertensives, to provide more evidence regarding how these factors influence the use of spot urine samples to estimate 24‐hour USE. Finally, more accurate methods of using the spot urine samples to evaluate individual 24‐hour USE are needed.

## 5 CONCLUSIONS

We found that the Kawasaki equation may have performed better than the other equations at Chinese Tibetan population level assessment, but none of these equations are suitable for use or perform well at the individual level. A more accurate method of using a spot urine sample to evaluate individual 24‐hour USE for Tibetans is needed.

## CONFLICT OF INTEREST

The authors declare that they have no competing interests to declare.

## ETHICS APPROVALS AND CONSENT TO PARTICIPATE

The research protocol was reviewed by the Medical Ethics committee of West China Hospital, Sichuan University. The study protocol conforms with the ethical guidelines of the Declaration of Helsinki. Informed written consent was obtained from patients before enrollment.

## CONSENT FOR PUBLICATION

All co‐authors and participants have given their consent for publication of this article in The Journal of Clinical Hypertension.

## AUTHOR CONTRIBUTIONS

Xin Zhang designed the study intellectual content, participated in original data acquisition, and wrote this initial manuscript. Runyu Ye carried out literature search and data acquisition. Xinran Li undertook the statistical analysis and participated in manuscript preparation. Qiling Gou, Zhipeng Zhang, and Zewong Zhuoma participated in clinical information acquisition. Rufeng Shi and Qingtao Meng participated in data acquisition of urine samples. Xiaoping Chen and Hengyu Zhang revised the manuscript for important intellectual content and languages. All authors read and approved the final manuscript.

## Data Availability

The datasets used and/or analyzed during the current study are available from the corresponding author upon reasonable request.
